# Exogenous ascorbic acid enhances drought tolerance in *Hypericum perforatum* L. by modulating antioxidant defense and osmotic adjustment

**DOI:** 10.1038/s41598-026-35931-6

**Published:** 2026-01-31

**Authors:** Fatemeh Asadi, Nematollah Etemadi, Rahim Amirikhah, Mohammad Reza Mosaddeghi, Hamed Aalipour

**Affiliations:** 1 Department of Horticulture, College of Agriculture, Isfahan University of Technology, Isfahan, 84156-83111 Iran; 2 Department of Horticulture, Faculty of Agriculture, University of Tabriz, Tabriz, Iran; 3 Department of Soil Science, College of Agriculture, Isfahan University of Technology, Isfahan, 84156-83111 Iran; 4 Department of Landscape Engineering, Faculty of Agriculture, University of Tabriz, Tabriz, Iran

**Keywords:** Antioxidant properties, Osmolytes, Oxidative stress, Stress mitigator, Water deficit, Biochemistry, Physiology, Plant sciences

## Abstract

Drought stress poses a significant threat to the cultivation of *Hypericum perforatum* L. (St. John’s wort), a valuable medicinal plant. While ascorbic acid (AsA) is a known mitigator of abiotic stress, its protective role and underlying mechanisms in *H. perforatum* remain unexplored. This study elucidates the biochemical and physiological basis of exogenous AsA-induced drought tolerance in *H. perforatum* subjected to full irrigation (100% FI), moderate (75% FI), and severe (50% FI) deficit irrigation. Severe drought significantly inhibited growth, reducing biomass, chlorophyll content, relative water content, and leaf area. Foliar application of AsA, particularly at 400 mg L^− 1^, markedly ameliorated these inhibitory effects. The AsA-mediated mitigation was mechanistically linked to a robust enhancement of the plant’s antioxidant defense system, evidenced by significantly increased activities of ascorbate peroxidase, catalase, and superoxide dismutase, alongside elevated levels of endogenous AsA, total phenolics, and osmolytes (proline and soluble carbohydrates). Consequently, AsA-pretreated plants exhibited substantially reduced oxidative damage, with lower levels of malondialdehyde and electrolyte leakage under severe stress. Our findings demonstrate that exogenous AsA fortifies drought tolerance in *H. perforatum* primarily by orchestrating a synergistic enhancement of osmotic adjustment and reactive oxygen species scavenging capacity. This effective and practical strategy highlights the potential of AsA application to sustain the production of this economically important species in water-limited environments.

**Supplementary Information:**

The online version contains supplementary material available at 10.1038/s41598-026-35931-6.

## Introduction

Global agricultural productivity faces escalating threats from abiotic stresses, with drought representing the most devastating constraint, responsible for approximately 48% of crop yield losses worldwide^1,2^. This challenge is intensifying due to climate change, increasing the frequency and severity of water deficit conditions, particularly in semi-arid and arid regions. Drought stress disrupts plant water homeostasis, triggering osmotic imbalance that impairs critical physiological processes including photosynthesis, stomatal conductance, and cellular metabolism^3,4^. A primary consequence of these disruptions is the accelerated generation of reactive oxygen species (ROS), resulting from impaired electron transport chains and reduced CO₂ assimilation^5,6^. While ROS function as signaling molecules at low concentrations, their overaccumulation causes oxidative damage to proteins, lipids, and nucleic acids, ultimately compromising cellular integrity and plant survival^7^.

To mitigate oxidative damage, plants have evolved sophisticated antioxidant defense systems comprising both enzymatic antioxidants such as catalase (CAT), superoxide dismutase (SOD), and ascorbate peroxidase (APX), and non-enzymatic components including ascorbate, proline, and phenolic^8–10^. The capacity to rapidly activate this integrated antioxidant network represents a crucial determinant of plant drought tolerance^11^.

Recent research has increasingly focused on employing exogenous phytoprotectants to enhance native defense mechanisms against environmental stresses. Among these, L-ascorbic acid (AsA, vitamin C) represents a particularly promising candidate due to its dual roles as both a fundamental metabolic regulator and a potent antioxidant^12–14^. AsA serves as an essential cofactor for numerous enzymes involved in photosynthesis, hormone biosynthesis, and cell division^15,16^, while simultaneously functioning as a primary substrate for ROS scavenging through the ascorbate-glutathione cycle^17^. Exogenous application of AsA has demonstrated efficacy in enhancing drought tolerance across various plant species, including pepper^18^, safflower^19^, and jute^20^, primarily through enhancing antioxidant capacity and osmoprotectant accumulation.

*Hypericum perforatum* L. (St. John’s wort) represents a species of considerable economic importance due to its pharmaceutical applications, particularly in the treatment of mild to moderate depression^21^, and its growing utilization in cosmetic and food industries^22^. Despite its agricultural value, the species’ response to drought stress remains inadequately characterized^23,24^, and the potential for enhancing its drought resilience through AsA application remains completely unexplored. Given the species-specific nature of stress responses and the variability in efficacy of exogenous protectants^17^, species-specific investigations are essential.

We hypothesized that exogenous AsA application would enhance drought tolerance in *H. perforatum* through coordinated enhancement of both enzymatic and non-enzymatic antioxidant systems, thereby reducing oxidative damage and maintaining physiological function under water deficit conditions. This study aimed to: (1) comprehensively characterize the physiological and biochemical responses of *H. perforatum* to progressive drought stress, and (2) elucidate the mechanistic basis of AsA-mediated protection through analysis of antioxidant capacity, osmolyte accumulation, and oxidative damage markers.

## Materials and methods

### Experimental conditions and plant materials

Five-leaf stage seedlings (4 weeks after sowing) of *H. perforatum* L. were sourced from a research field in Mahmoudabad, Iran (51° 35′ 06′′ E, 32° 47′ 47.8′′ N). Each seedling was transplanted into a plastic pot (18 cm diameter ×17 cm height) containing 2.0 kg of a homogenized substrate composed of soil, leaf compost, and sand in a 1:2:2 volumetric ratio. The substrate was characterized as a loam texture with a gravimetric permanent wilting point (PWP) of 14% and a field capacity (FC) of 31%. The physical and chemical characteristics of the substrate are shown in Table 1.

**Table 1:** Physical and chemical characteristics of the substrate.

Sand (%)	Silt (%)	Clay (%)	Texture	F.C. (%)	PWP (%)	pH	EC (dS m⁻¹)
35.92	43.28	20.8	loam	31	14	7.5	1.78

The experiment was conducted between February and June, 2021, in a controlled greenhouse with a clear glass cover at the Isfahan University of Technology, Iran (51° 39′ 40′′ E, 32° 38′ 30′′ N; altitude 1600 m). Environmental conditions were maintained at 22 ± 4 °C, 60 ± 5% relative humidity, under natural light photoperiods and intensity. No supplemental lighting was used. All plants were maintained under optimal watering and nutritional conditions for an 8-week establishment period prior to the initiation of experimental treatments. The NPK fertilizer (20-20-20) was applied as fertigation (1000 mg L^− 1^) at third week after transplanting.

The study was arranged in a 3 × 3 factorial experiment based on a completely randomized design (CRD) in four replications with two pots per replication. Eight uniform plants were used per treatment. The two factors were: (1) Drought Stress: three irrigation regimes—full irrigation (100% of FI, control), moderate deficit irrigation (75% of FI), and severe deficit irrigation (50% of FI); and (2) Ascorbic Acid (AsA) Treatment: three concentrations—0 (control), 200, and 400 mg L^− 1^.

### Ascorbic acid application and drought stress imposition

Prior to drought induction, plants were pretreated with AsA via foliar spray at vegetative growth stage. Solutions of 200, and 400 mg L^− 1^ AsA (in deionized water) were applied to runoff twice at 7-day intervals. These concentrations were selected according to the previous studies^19,25^. AsA solutions containing 0.1% (v/v) Tween-20, as a non-ionic surfactant, were prepared freshly in deionized water. The pH of the AsA solution was adjusted to 5.5 to ensure improved foliar uptake. Control plants (0 mg L^− 1^ AsA) were sprayed with deionized water containing 0.1% Tween 20. All AsA solutions were sprayed in the early morning with an average volume of 3 mL per plant.

Following the second AsA application, the drought stress treatments were initiated. The irrigation volumes for each treatment were precisely calculated based on soil water properties. The total available water (TAW, cm⁻³) and the height of TAW (h_TAW_, mm) in the pot were calculated as follows^26^:

1 $$\:\mathrm{T}\mathrm{A}\mathrm{W}=\frac{\left(\mathrm{F}\mathrm{C}-\mathrm{P}\mathrm{W}\mathrm{P}\right)}{100}\times\:\frac{{\rho\:}_{b}}{{\rho\:}_{w}}$$

2 $$\:{\mathrm{h}}_{\mathrm{T}\mathrm{A}\mathrm{W}}=\mathrm{T}\mathrm{A}\mathrm{W}\times\:\mathrm{D}=\frac{\left(\mathrm{F}\mathrm{C}-\mathrm{P}\mathrm{W}\mathrm{P}\right)}{100}\times\:\frac{{\rho\:}_{b}}{{\rho\:}_{w}}\times\:\mathrm{D}$$

Where PWP is permanent wilting point (g 100 g^− 1^), FC is field capacity (g 100 g^− 1^), ρ_b_ and ρ_w_ are soil bulk density and water density (g cm^− 3^), respectively, and D is the depth of the root zone equal to the soil height in the pot (mm).

The irrigation threshold for the FI treatment was set at 50% maximum allowable depletion (MAD). The height of readily available water (h_RAW_, mm) and the soil water content at irrigation (θirr, g 100 g⁻¹) were calculated as:

3 $$\:{\mathrm{h}}_{\mathrm{R}\mathrm{A}\mathrm{W}}={\mathrm{h}}_{\mathrm{T}\mathrm{A}\mathrm{W}}\times\:\mathrm{M}\mathrm{A}\mathrm{D}$$

4 $$\:{{\uptheta\:}}_{\mathrm{i}\mathrm{r}\mathrm{r}}=\mathrm{F}\mathrm{C}-\mathrm{M}\mathrm{A}\mathrm{D}\times\:(\mathrm{F}\mathrm{C}-\mathrm{P}\mathrm{W}\mathrm{P})$$

The calculated h_RAW_ was 7.08 mm (180 ml) per pot for the 100% FI treatment. The moderate (75% FI) and severe (50% FI) deficit irrigation treatments received 75% and 50% of this volume, respectively. The irrigation schedule was determined by daily monitoring of pot weight and soil water content using a probe to ensure θ_irr_ was reached before each watering event.

### Assessment of growth, biochemical, and physiological parameters

Following the 8-week treatment period, a comprehensive evaluation of growth, biochemical, and physiological parameters was conducted. For subsequent analyses, freshly collected leaf samples were immediately flash-frozen in liquid nitrogen and kept at -80 °C.

#### Morphological characteristics

At harvest, the shoot length of each plant was measured. Plants were then carefully harvested from their pots, and the root system was gently rinsed with tap water to clean off adhering substrate. The following morphological traits were recorded: root length, root volume, and the fresh weight of shoots and roots^27^. For dry weight determination, shoot and root samples were oven-dried at 70 °C for 72 h to a constant weight. Leaf area was measured on five randomly selected fully expanded leaves per plant using an leaf area meter (Win Area-UT-11, Iran)^28^.

#### Physiological measurements

##### Relative water content (RWC)

Leaf RWC was determined according to the method of Smart and Bingham (1974)^29^. Briefly, fresh weight (FW) was recorded immediately after collecting five young, fully expanded leaves. The leaves were then floated in distilled water for 4 h at room temperature to achieve full turgor, after which turgid weight (TW) was calculated. Finally, the leaves were oven-dried at 70 °C for 72 h to determine dry weight (DW). RWC was calculated using the formula:

5 $$\:\mathrm{R}\mathrm{W}\mathrm{C}=\frac{\mathrm{F}\mathrm{W}-\mathrm{D}\mathrm{W}}{\mathrm{T}\mathrm{W}-\mathrm{D}\mathrm{W}}\times\:100$$

##### Chlorophyll and cartenoid contents

The contents of chlorophyll *a* (Chl *a*), chlorophyll *b* (Chl *b*), total chlorophyll (Chl T), and total carotenoids were quantified following the spectrophotometric method of Lichtenthaler and Buschmann (2001)^30^. Briefly, 0.1 g of fresh leaf tissue was homogenized in 10 mL of 100% acetone and centrifuged. The absorbance of the extract was recorded at 644.8, 661.6, and 470 nm using a UV-160 A spectrophotometer (Shimadzu, Japan). Pigment concentrations were calculated according to the following equations:

6 $$\:Chl\:a=11.24\left(A661.6\right)-2.04\left(A644.8\right)$$

7 $$\:Chl\:b=20.13\:\left(A644.8\right)-4.19\left(A661.6\right)$$

8 $$\:Chl\:\:T(\mu\:g/mL)\:=7.05\:\left(A661.6\right)+18.09\left(A644.8\right)$$

9 $$\:C\:x+c\:(\mu\:g/mL)\:=(1000\:\left(A470\right)-1.90\left(Ca\right)-63.14\left(Cb\right))/214$$

Where Chl T is concentration of total chlorophyll (µg/mL), and Cx + c is the carotenoids concentration (µg/mL).

##### Chlorophyll fluorescence

The maximum quantum efficiency of photosystem II (PSII) was determined as the Fv/Fm ratio using a portable Plant Efficiency Analyzer (PEA, Hansatech Instruments Ltd., UK). Fully expanded leaves were dark-adapted for 30 min prior to measurement. The Fv/Fm index was measured between 11:00 and 12:00 a.m. following established protocols^31^.

#### Oxidative stress indicators

##### Electrolyte leakage (EL)

EL was determined according to Lutts et al. (1996)^32^. Leaf discs were submerged in distilled water and incubated at 25 °C with shaking (100 rpm) for 12 h. Measurement of initial electrical conductivity (EC₁) was performed with a conductivity meter (Model CC-501). This was followed by autoclaving of the samples at 120 °C for 20 min and subsequent measurement of final conductivity (EC₂) upon cooling to ambient temperature. EL was calculated as:

10 $$\:\mathrm{E}\mathrm{L}\:\left(\mathrm{\%}\right)=\left(\frac{\mathrm{E}\mathrm{C}1}{\mathrm{E}\mathrm{C}2}\right)\times\:100$$

##### Malondialdehyde (MDA) content

MDA content, a marker of lipid peroxidation, was quantified via thiobarbituric acid reactive substances (TBARS) assay^33^. Briefly, 0.1 g leaf tissue was homogenized in 2 mL of 1.5% (w/v) trichloroacetic acid (TCA) and centrifuged at 15,339 × g for 15 min. Two milliliters of supernatant were mixed with 2 mL of 20% TCA containing 0.5% (w/v) thiobarbituric acid (TBA). The reaction mixture was incubated in a boiling water bath for 30 min, rapidly cooled, and centrifuged (7,826 × g, 10 min). Absorbance of the final supernatant was measured at 532 nm and 600 nm. MDA concentration was calculated with an extinction coefficient of 155 mM⁻¹ cm⁻¹.

#### Osmolytes contents

##### Proline content

Proline was quantified by Bates et al. (1973)^34^ procedure. A total of 200 mg of fresh leaf samples was homogenized in 3% sulphosalicylic acid. Subsequently, 2 ml of the supernatant was combined with 2 ml of ninhydrin reagent and 2 ml of glacial acetic acid. The reaction mixture was maintained in a water bath at 100 °C for 1 h. After cooling, the reaction mixtures were extracted with 4 ml of toluene. The absorbance readings were taken at 520 nm.

##### Total soluble carbohydrate

Total soluble carbohydrate was measured spectrophotometrically using the anthrone method^35^. Fresh leaf tissues (200 mg) were homogenized in 10 mL of 80% ethanol. Supernatants were centrifuged for 15 min at 5000 rpm after being in a water bath at 70 °C for 10 min. Subsequently, a 100 µl of the supernatant was mixed with anthrone reagent and heated in a water bath at 100 °C for 10 min. The absorbance of the resulting mixture was recorded at 630 nm using a Spectrophotometer (Shimadzu, Japan).

#### Non-enzymatic antioxidants

##### Endogenous ascorbic acid (AsA) content

Endogenous AsA content was determined spectrophotometrically according to Mukherjee and Choudhuri (1983)^36^. Fresh leaf tissue (0.5 g) was homogenized in 10 mL of 6% TCA. The extract (4 mL) was mixed with 2 mL of dinitrophenyl hydrazine reagent (containing one drop of thiourea solution), incubated in a water bath (100 °C) for 15 min, and cooled to room temperature. Five milliliters of 80% H₂SO₄ were added, and absorbance was measured at 530 nm. Quantification was based on an AsA standard curve.

##### Total phenolic content (TPC)

TPC was determined with the Folin-Ciocalteu assay^37^. Fresh leaf tissue (0.1 g) was homogenized in 5 mL of 80% acetone and centrifuged (10,000 × g, 10 min). A 0.1 mL aliquot of supernatant was combined with 1 mL of Folin-Ciocalteu reagent and vigorously shaken. After 5 min, 5 mL of 20% sodium carbonate solution was added, and the final volume was adjusted to 10 mL with distilled water. After 60 min of incubation, absorbance was read at 750 nm. TPC was expressed as mg of gallic acid equivalents per gram of fresh weight.

##### DPPH (2,2-diphenyl-1-picrylhydrazyl) free radical scavenging assay

The antioxidant activity of the methanolic plant leaf extracts was evaluated using the DPPH radical scavenging assay^38^. Briefly, 100 µL of each the plant extract was mixed with 3 mL of a 0.1 mM methanolic DPPH solution in test tubes. A control was prepared by replacing the extract with the same volume (100 µL) of methanol, as a solvent. The mixtures were vortexed thoroughly and incubated in the dark at room temperature for 30 min to prevent photodegradation of DPPH. After incubation, the absorbance was measured at 517 nm using a UV‑Vis spectrophotometer (Shimadzu, Japan). The radical scavenging activity was calculated as a percentage inhibition using the formula:

11 $$\:\mathrm{D}\mathrm{P}\mathrm{P}\mathrm{H}\:\mathrm{s}\mathrm{c}\mathrm{a}\mathrm{v}\mathrm{e}\mathrm{n}\mathrm{g}\mathrm{i}\mathrm{n}\mathrm{g}\:\mathrm{a}\mathrm{c}\mathrm{t}\mathrm{i}\mathrm{v}\mathrm{i}\mathrm{t}\mathrm{y}\:\left(\mathrm{\%}\right)=\left(\frac{({A}_{\_\mathrm{c}\mathrm{o}\mathrm{n}\mathrm{t}\mathrm{r}\mathrm{o}\mathrm{l}}-\mathrm{A}\_\mathrm{s}\mathrm{a}\mathrm{m}\mathrm{p}\mathrm{l}\mathrm{e})}{\mathrm{A}\_\mathrm{c}\mathrm{o}\mathrm{n}\mathrm{t}\mathrm{r}\mathrm{o}\mathrm{l}}\right)\times\:100$$

Where A__control_ is the absorbance of the DPPH solution with solvent, A__sample_ is the absorbance of DPPH with extract.

#### Antioxbidant enzyme assays

Enzyme extracts were prepared by homogenizing 0.1 g leaf tissue in 1 mL of 100 mM potassium phosphate buffer (pH 7.0) containing 0.5% (v/v) Triton X-100 and 1.0% (w/v) polyvinylpyrrolidone. Homogenates were centrifuged at 17,608 × g for 15 min at 4 °C, and the supernatant was used for enzyme assays. All enzyme activities were expressed per mg protein.

##### Catalase (CAT; EC 1.11.1.6)

CAT activity was assayed according to Aebi (1983)^39^ by monitoring the decomposition of H₂O₂ at 240 nm (ε = 39.4 mM⁻¹ cm⁻¹). The reaction mixture contained 100 mM phosphate buffer (pH 7.0), 15 mM H₂O₂, and 50 µL enzyme extract in a total volume of 3 mL.

##### Ascorbate peroxidase (APX; EC 1.11.1.11)

APX activity was determined following Nakano and Asada (1981)^40^ by measuring the oxidation of ascorbate at 290 nm (ε = 2.8 mM⁻¹ cm⁻¹). The reaction mixture contained 100 mM phosphate buffer (pH 7.0), 0.5 mM H₂O₂, 0.1 mM EDTA, 5 mM ascorbate, and 50 µL enzyme extract.

##### Superoxide dismutase (SOD; EC 1.15.1.1)

SOD activity was assayed according to Giannopolitis and Ries (1977)^41^ based on the inhibition of photochemical reduction of nitroblue tetrazolium (NBT). The reaction mixture contained 100 mM phosphate buffer (pH 7.8), 75 nM EDTA, 13 mM L-methionine, 50 µM NBT, 0.02 mM riboflavin, and 50 µL enzyme extract. Tubes were illuminated with fluorescent lamps (5000 lx) for 15 min, and absorbance was measured at 560 nm. One unit of SOD activity was defined as the amount of enzyme that caused 50% inhibition of NBT reduction.

### Statistical analysis

All data are presented as mean ± standard error (SE) of four independent biological replicates (*n* = 4). Data were subjected to two-way analysis of variance (ANOVA) using SAS software (SAS Institute, 2001) to examine the effects of drought stress, AsA treatment, and their interaction. Assumptions of normality (Kolmogorov-Smirnov or Shapiro-Wilk tests) and homogeneity of variance (Levene’s or Bartlett’s tests) were verified prior to ANOVA. When significant interactions were detected (*p* < 0.05), means were separated using Tukey’s honestly significant difference (HSD) test at α = 0.05. When the interaction was not significant, the main effects of drought and AsA were analyzed independently. In graphical presentations, significant differences (*p* < 0.05) for main effects are denoted with separate lettering: uppercase letters for drought levels and lowercase letters for AsA treatments. Multivariate analysis including hierarchical clustering with heatmap visualization was performed using Chiplot (https://www.chiplot.online/), and principal component analysis (PCA) was conducted using Statgraphics Centurion version 19.

## Results

### Ascorbic acid ameliorates drought-induced suppression of shoot and root biomass

**Fig. 1:**
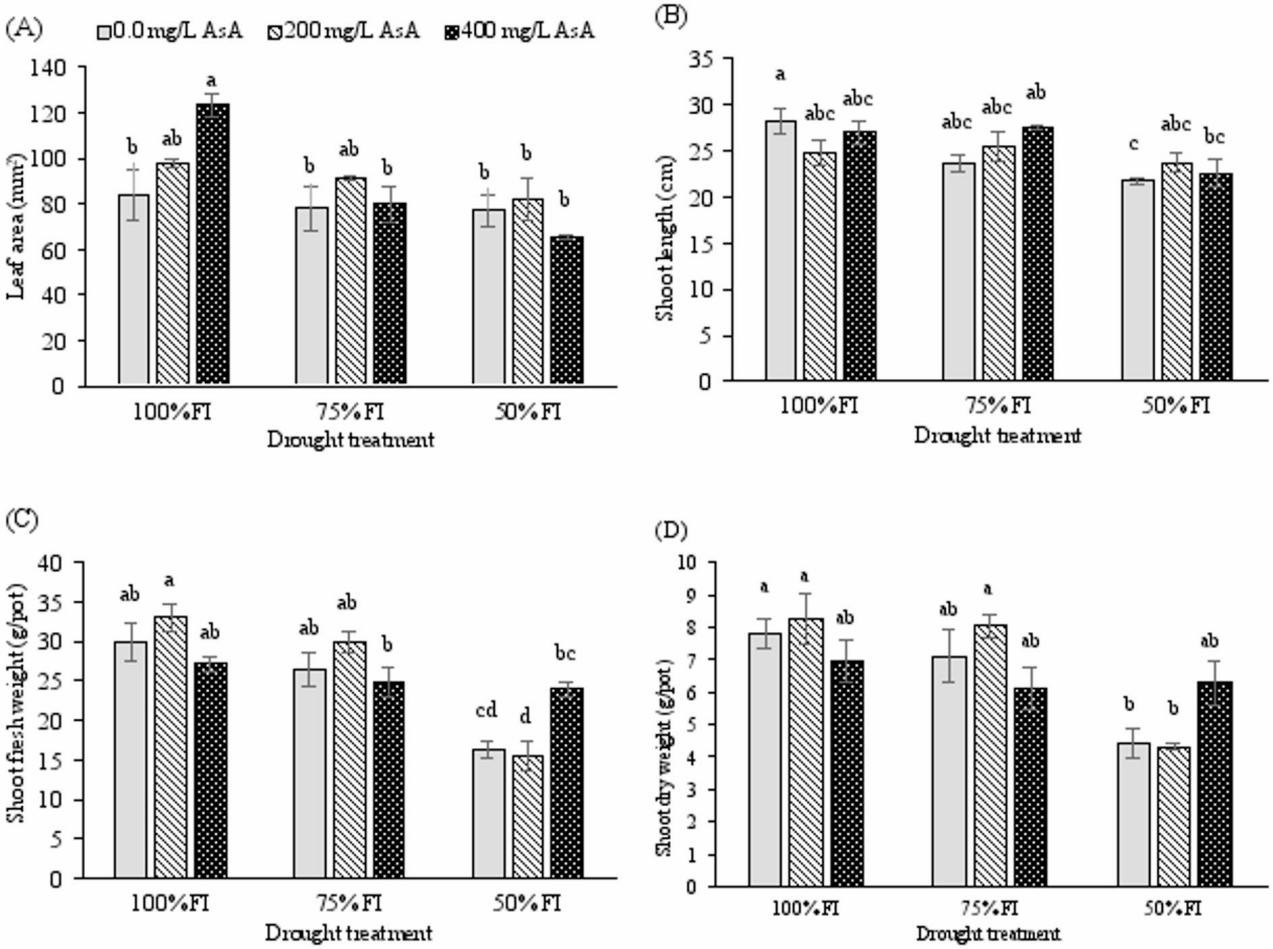
Changes in leaf area, shoot length, and shoot fresh and dry weights of *H. perforatum* as affected by the interaction of drought stress and ascorbic acid (AsA). FI: full irrigation; 75% FI: moderate deficit irrigation; and 50% FI: severe deficit irrigation. The bars with at least one similar letter are not significantly different (*p* < 0.05) according to Tukey’s test.

The interaction between ascorbic acid (AsA) treatment and drought stress significantly influenced the leaf area of *H. perforatum* plants (*p* < 0.01; Table 2). Under full irrigation (FI), AsA application, particularly at 400 mg L⁻¹, significantly increased leaf area compared to the untreated control, with a maximum value of 123.12 mm² (Fig. 1A). However, under moderate (75% FI) and severe (50% FI) deficit irrigation, the positive effect of AsA on leaf area was attenuated, and no significant difference was observed between AsA-treated and untreated plants at these stress levels.

**Table 2:** Mean squares from ANOVA for morphophysiological and biochemical characters of *H. perforatum* as affected by drought stress (100% full Irrigation, 75% FI, 50%FI) and ascorbic acid (AsA).

	Sources of variation			
Traits	Drought (D)	Ascordic acid (AsA)	D×AsA	Error
LA	2258.5**	420.3^ns^	823.9**	196.8
SL	51.31**	4.60 ^ns^	14.05 *	5.19
SFW	425.36**	10.82 ^ns^	69.22**	11.11
SDW	16.98**	0.7^ns^	8.17**	1.4
RL	4.86 ^ns^	0.18 ^ns^	1.95 ^ns^	3.71
RV	489.05**	14.31 ^ns^	65.29 ^ns^	33.67
RFW	513.93**	5.52 ^ns^	8.30 ^ns^	4.48
RDW	7.86 **	0.26 ^ns^	1.23 *	0.39
ChlT	0.982*	0.043 ^ns^	1.602**	0.266
CXC	0.107**	0.004 ^ns^	0.091**	0.008
FV/FM	0.0049**	0.0003 ^ns^	0.0012**	0.0002
RWC	1280.38**	213.71*	47.86 ^ns^	51.96
TSC	10.52**	0.26 ^ns^	2.32 ^ns^	1.11
PRO	408.07**	12.78 ^ns^	12.88^*^	4.60
EL	1239.84**	53.34 ^ns^	11.75 ^ns^	19.17
MDA	2.21**	1.81**	0.16 *	0.05
AsA	0.24**	0.25**	0.01 ^ns^	0.01
TPC	7.73 **	2.35 **	0.42 ^ns^	0.28
DPPH	147.09*	346.56**	68.41 ^ns^	31.55
CAT	43138.1**	81295.8**	11669.2**	2062.4
APX	0.0189**	0.0287**	0.0066**	0.0008
SOD	7114.3**	2665.8**	2353.5**	394.0

**: *p* < 0.01; *: *p* < 0.05; ns: not significant. Leaf area (LA), shoot length (SL), shoot fresh weight (SFW), shoot dry weight (SDW), root length (RL), root dry weight (RDW), root fresh weight (RFW), root volume (RV), total chlorophyll (ChlT), carotenoid (CXC), Fv/Fm index, relative water content (RWC), total soluble carbohydrate content (TSC), proline (PRO), electrolyte leakage (EL), malondialdehyde (MDA), ascorbate (AsA), total phenolic content (TPC), DPPH radical scavenging, catalase (CAT), ascorbate peroxidase (APX), and superoxide dismutase (SOD).

Shoot length (SL) was significantly affected by drought stress (*p* < 0.001) and its interaction with AsA treatment (*p* < 0.05). Severe drought stress (50% FI) caused a maximum reduction of 23.6% in SL in untreated plants (Fig. 1B). In contrast, AsA application mitigated this reduction, as no significant differences in SL were observed among AsA-treated plants (200 and 400 mg L⁻¹) across different irrigation regimes.

**Fig. 2:**
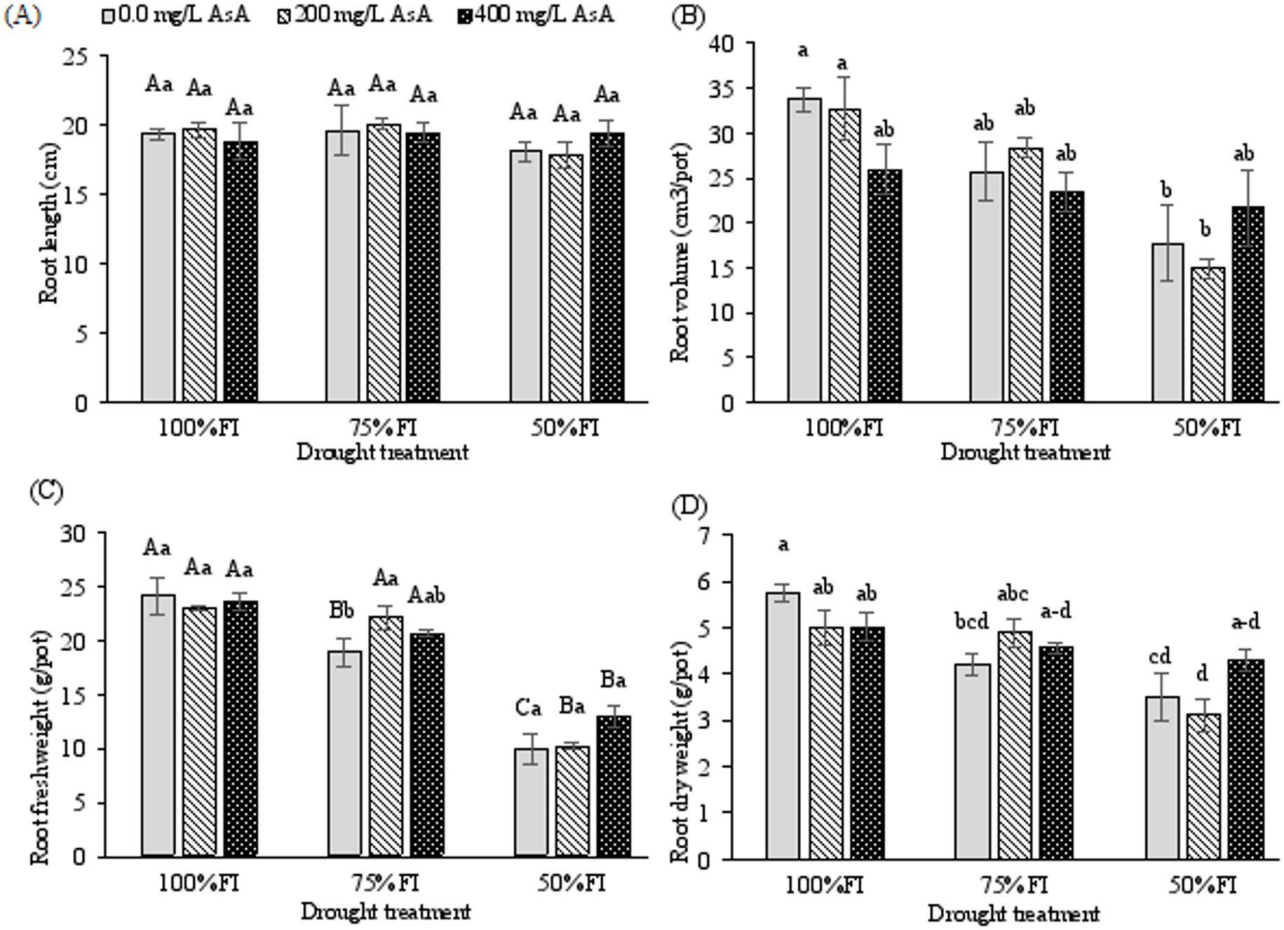
Changes in root length, root volume, and root fresh and dry weights of *H. perforatum* as affected by the interaction of drought stress and ascorbic acid (AsA). FI: full irrigation; 75% FI: moderate deficit irrigation; and 50% FI: severe deficit irrigation. The bars with at least one similar letter are not significantly different (*p* < 0.05) according to Tukey’s test. When the interaction between drought and AsA treatment was not significant (*p* > 0.05), the significant main effects were interpreted separately. In the corresponding figures, different uppercase letters indicate significant differences among drought levels, and different lowercase letters indicate significant differences between AsA treatments (Tukey’s HSD test, *p* < 0.05).

A significant interaction between drought stress and AsA treatment was also observed for shoot fresh weight (SFW) and shoot dry weight (SDW) (*p* < 0.001 for both; Table 2). Both SFW and SDW exhibited a general decline with increasing drought intensity (Fig. 1C, D). While severe deficit irrigation (50% FI) significantly reduced the biomass of untreated plants and those treated with 200 mg L⁻¹ AsA, plants treated with 400 mg L⁻¹ AsA showed remarkable resilience. Under severe deficit irrigation (50% FI), their SFW and SDW values were not significantly different from those of well-watered control plants (FI, 0 mg L⁻¹ AsA), demonstrating a near-complete mitigation of the drought-induced biomass reductionDrought stress significantly impaired root system development, with severe deficit irrigation (50% FI) exerting the most pronounced negative effects on root fresh weight (RFW), root volume (RV), and root dry weight (RDW) (*p* < 0.001 for all parameters; Fig. 2B-D). The highest root volume (33.66 cm³) was recorded under full irrigation conditions, while the most substantial reduction occurred under severe drought stress with 200 mg L⁻¹ AsA treatment (14.83 cm³).

Notably, ascorbic acid application modulated root responses to water deficit in a concentration-dependent manner. Under moderate deficit irrigation (75% FI), increasing AsA concentrations showed a positive trend in RFW enhancement. A significant interaction between drought stress and AsA treatment was observed specifically for RDW (*p* = 0.030), indicating that the efficacy of AsA in maintaining root biomass was contingent upon stress severity.

Root length (RL) exhibited particular sensitivity to AsA treatment. However, there were no significant differences between RL of AsA-treated and untreated plants (Table 2 Fig. 2). These findings collectively demonstrate that foliar AsA application, particularly at optimal concentrations, can partially mitigate the inhibitory effects of drought stress on root system development in *H. perforatum*.

### AsA application maintains leaf water status and photosynthetic pigments under drought

**Fig. 3:**
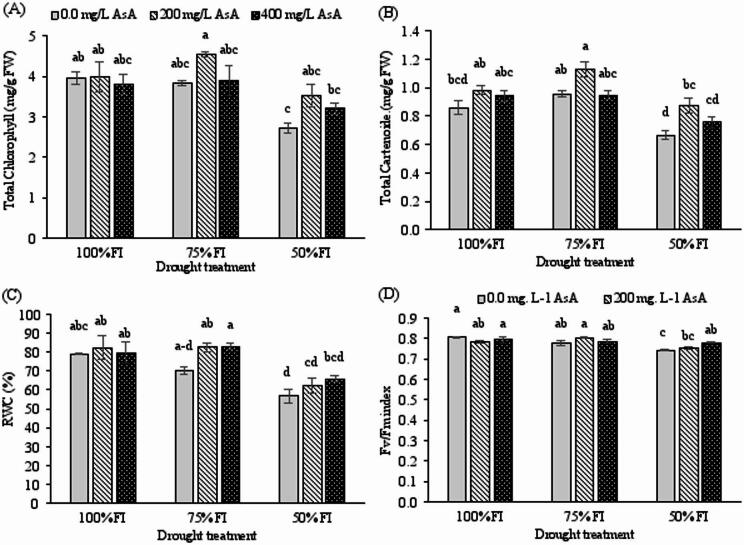
Changes in total chlorophylls, total cartenoids, relative water content (RWC), and Fv/Fm index of *H. perforatum* as affected by the interaction of drought stress and ascorbic acid (AsA). FI: full irrigation; 75% FI: moderate deficit irrigation; and 50% FI: severe deficit irrigation. The bars with at least one similar letter are not significantly different (*p* < 0.05) according to Tukey’s test.

Drought stress significantly reduced photosynthetic pigment content, with total chlorophyll and carotenoid concentrations showing pronounced decreases under 50% FI conditions (*p* < 0.001; Table 2). The most severe reductions occurred in untreated plants under 50% FI irrigation, with total chlorophyll declining by 31.2% and carotenoids by 22.3% compared to well-watered controls (Fig. 3A, B).

Exogenous AsA application effectively mitigated these drought-induced reductions in photosynthetic pigments. Plants treated with 400 mg L⁻¹ AsA maintained significantly higher chlorophyll and carotenoid levels under severe drought stress compared to untreated stressed plants (Fig. 3A, B), demonstrating the protective role of ascorbic acid in preserving photosynthetic apparatus integrity.

**Fig. 4:**
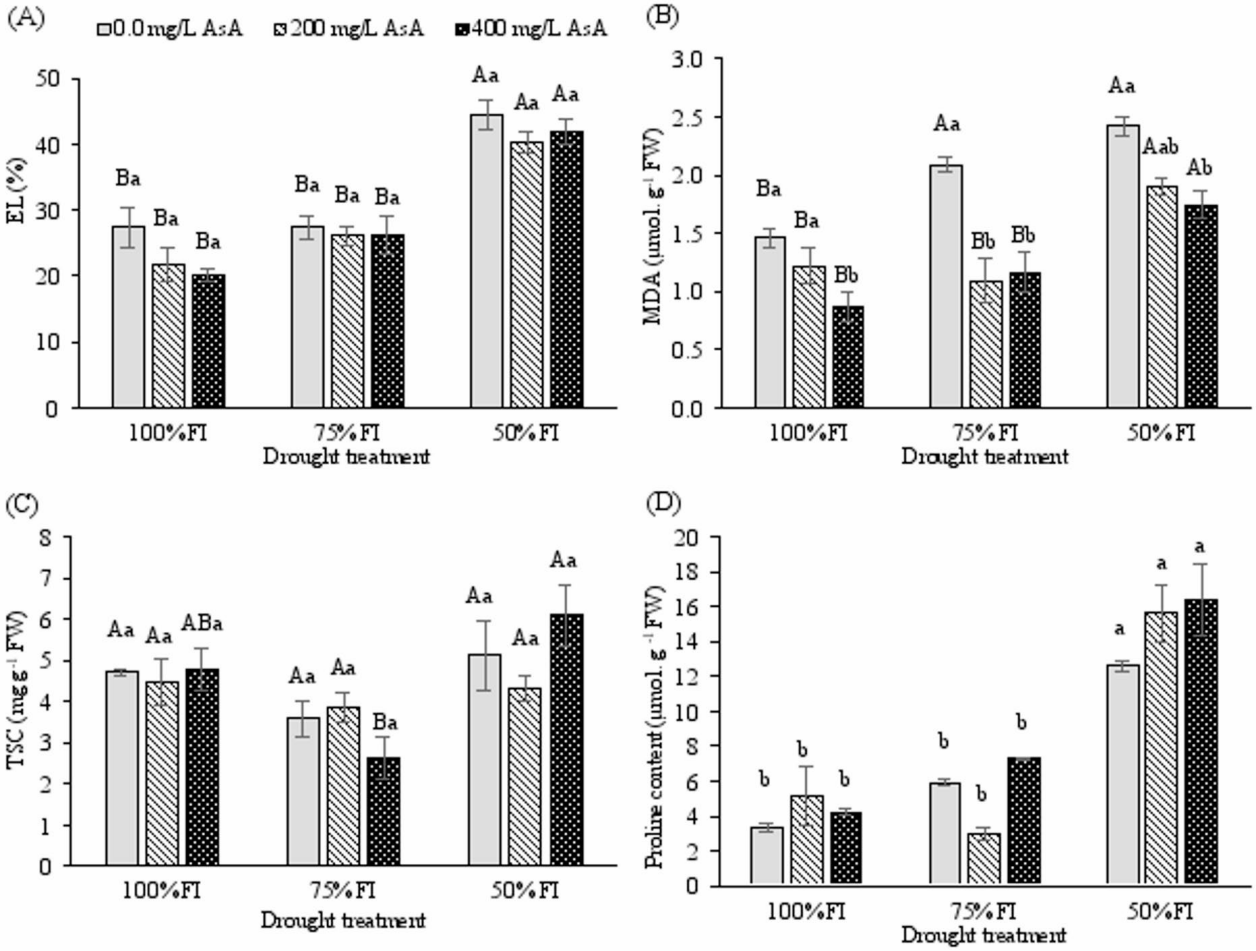
Changes in electrolyte leakage (EL), malondialdehyde (MDA) contents, total soluble carbohydrate content (TSC), and proline content of *H. perforatum* as affected by the interaction of drought stress and ascorbic acid (AsA). FI: full irrigation; 75% FI: moderate deficit irrigation; and 50% FI: severe deficit irrigation. The bars with at least one similar letter are not significantly different (*p* < 0.05) according to Tukey’s test. When the interaction between drought and AsA treatment was not significant (*p* > 0.05), the significant main effects were interpreted separately. In the corresponding figures, different uppercase letters indicate significant differences among drought levels, and different lowercase letters indicate significant differences between AsA treatments (Tukey’s HSD test, *p* < 0.05).

Drought stress significantly reduced RWC in a severity-dependent manner (*p* < 0.001), while exogenous AsA application partially mitigated this decline (*p* < 0.05; Table 2). Severe deficit irrigation (50% FI) caused a maximum RWC reduction of 28.2% in untreated plants (Fig. 4A). Although AsA treatment (200 mg L⁻¹) showed limited protection under severe stress, the highest concentration (400 mg L⁻¹) demonstrated a clear trend toward maintaining tissue hydration.

### Foliar AsA preserves photosynthetic efficiency under water-deficit conditions

The maximum quantum efficiency of PSII (Fv/Fm ratio) was significantly affected by both drought stress (*p* < 0.001) and its interaction with AsA treatment (*p* < 0.01; Table 2). Water deficit caused progressive reduction in Fv/Fm values, indicating impaired photochemical efficiency. However, AsA pretreatment, particularly at 400 mg L⁻¹, significantly alleviated this decline (Fig. 3C). Under severe drought conditions, AsA-treated plants maintained Fv/Fm values comparable to those of moderately stressed plants, while untreated plants showed the most substantial photochemical impairment.

### Ascorbic acid mitigates oxidative membrane damage caused by drought stress

Oxidative stress indicators revealed substantial membrane damage under drought conditions. EL increased progressively with drought intensity, reaching 63% above control values in severely stressed untreated plants (Fig. 4B). AsA application, particularly at 400 mg L⁻¹, reduced EL by approximately 5.71% compared to untreated plants under severe drought, indicating enhanced membrane stability.

Lipid peroxidation, measured as MDA content, showed significant responses to both drought stress (*p* < 0.001) and AsA treatment (*p* < 0.001), with a notable interaction effect (*p* < 0.05; Table 2). MDA accumulation began at moderate stress levels (30% increase at 75% FI) and peaked under severe drought (Fig. 4C). Crucially, 400 mg L⁻¹ AsA treatment reduced MDA content by 28% compared to untreated plants under severe stress, demonstrating significant protection against oxidative membrane damage.

### AsA enhances osmotic adjustment through proline and sugar accumulation

Drought stress triggered substantial accumulation of osmolytes, with proline and soluble carbohydrates showing distinct response patterns. Soluble carbohydrate content increased under drought stress primarily driven by the severe (50% FI) treatment. While the 400 mg L⁻¹ AsA-treated plants under severe drought maintained numerically higher carbohydrate levels than untreated plants, this difference was not statistically significant (Fig. 4D). This coordinated accumulation of compatible solutes suggests that AsA enhances osmotic adjustment capacity in drought-stressed plants.

Proline content exhibited the most dramatic increase, rising by 284% in untreated plants under severe drought compared to well-watered controls (Fig. 4E). AsA application further enhanced this accumulation, with 400 mg L⁻¹ treatment resulting in 30% increase over untreated plants under severe drought stress - the highest observed concentration.

### AsA treatment boosts the non-enzymatic antioxidant pool

**Fig. 5:**
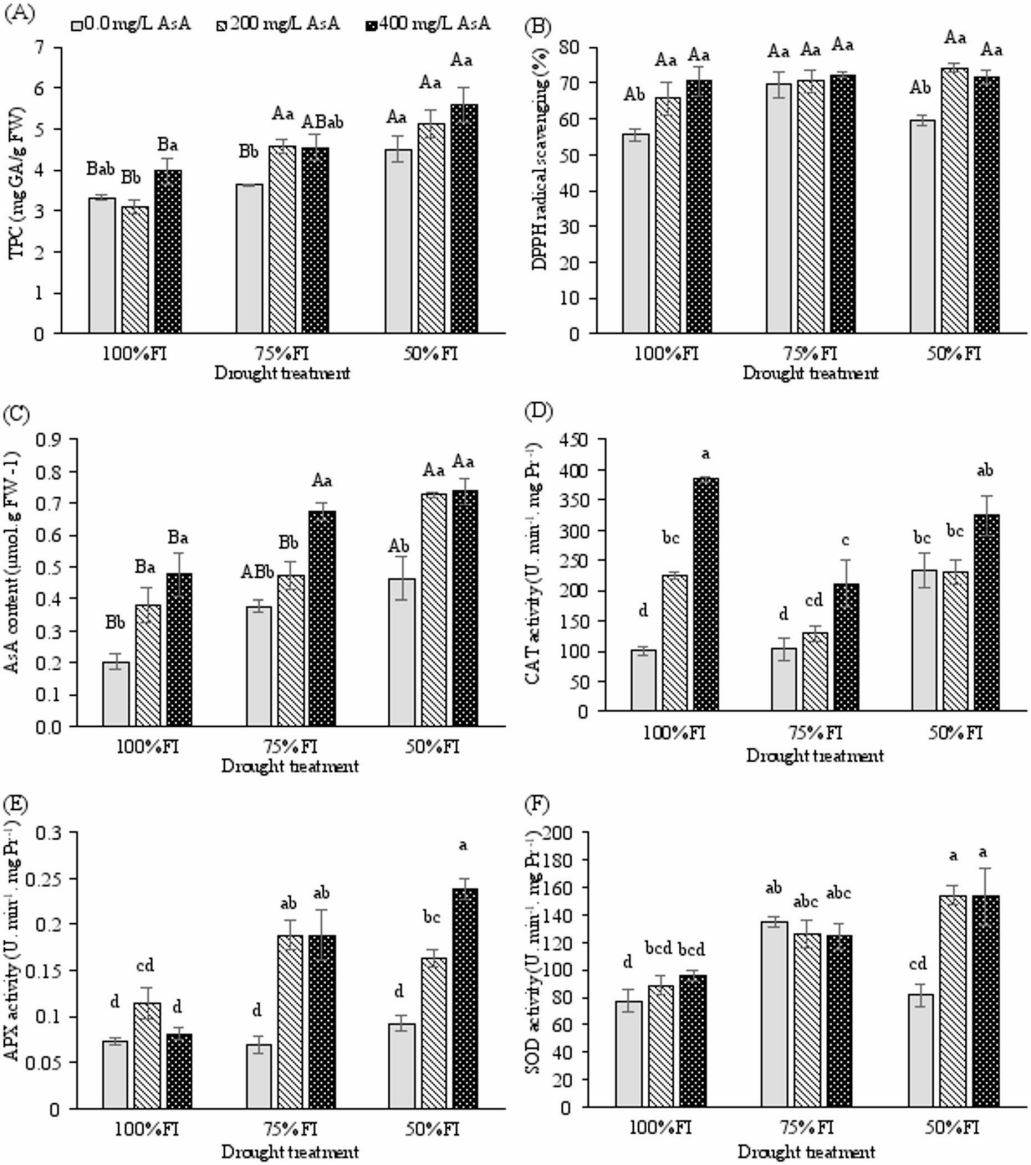
Changes in total phenolic content (TPC), DPPH radical scavenging (%), endogenous ascorbic acid (AsA) contents, catalase (CAT), ascorbate peroxidase (APX), and superoxide dismutase (SOD) of *H. perforatum* as affected by the interaction of drought stress and ascorbic acid (AsA). FI: full irrigation; 75% FI: moderate deficit irrigation; and 50% FI: severe deficit irrigation. The bars with at least one similar letter are not significantly different (*p* < 0.05) according to Tukey’s test. When the interaction between drought and AsA treatment was not significant (*p* > 0.05), the significant main effects were interpreted separately. In the corresponding figures, different uppercase letters indicate significant differences among drought levels, and different lowercase letters indicate significant differences between AsA treatments (Tukey’s HSD test, *p* < 0.05).

Drought stress significantly enhanced the biosynthesis of protective compounds, with total phenolic content (TPC) showing a substantial increase under water deficit conditions (*p* < 0.001; Fig. 5A). The interaction between drought intensity and AsA treatment revealed a complex response pattern, where moderate drought alone did not significantly alter TPC, but severe drought (50% FI) triggered remarkable phenolic accumulation. Exogenous AsA application at 200 and 400 mg L⁻¹ under severe drought stress further amplified this response, increasing TPC by 54.22% and 68.07%, respectively, compared to untreated plants at 100% FI conditions.

The antioxidant capacity, as measured by DPPH radical scavenging activity, was significantly enhanced by both drought stress (*p* < 0.01) and AsA treatment (*p* < 0.001; Table 2). Under severe deficit irrigation, AsA-treated plants exhibited substantially higher antioxidant activity compared to untreated controls (Fig. 5B), indicating that exogenous AsA application potentiated the plant’s innate antioxidant defense mechanisms under stress conditions.

Endogenous AsA content was profoundly influenced by both exogenous AsA application and drought stress (*p* < 0.001; Table 2). Under optimal irrigation conditions (100% FI), foliar application of 200 and 400 mg L⁻¹ AsA significantly elevated endogenous AsA levels by 38% and 72%, respectively, compared to untreated controls (Fig. 5C). This effect was particularly pronounced under drought stress, where AsA-treated plants maintained 2.3- to 2.8-fold higher endogenous AsA levels compared to untreated plants under severe water deficit. The enhanced endogenous AsA pool in treated plants suggests improved capacity for ROS scavenging and redox homeostasis maintenance under drought conditions.

### Ascorbic acid primes the activity of key antioxidant enzymes

The activities of key antioxidant enzymes exhibited significant responses to both drought stress and AsA treatment (*p* < 0.001 for all enzymes; Table 2), with notable interaction effects indicating the synergistic enhancement of the antioxidant system.

Under full irrigation conditions, exogenous AsA application markedly stimulated CAT activity, with 200 and 400 mg L⁻¹ treatments increasing enzyme activity by 124.1% and 282.6%, respectively, compared to untreated controls (Fig. 5D). This priming effect persisted under drought stress, where plants treated with 400 mg L⁻¹ AsA maintained CAT activity levels 38.62% higher than untreated plants under severe water deficit, demonstrating the crucial role of AsA in enhancing H₂O₂ detoxification capacity.

APX activity showed a progressive enhancement with increasing drought intensity (Fig. 5E). While moderate stress (75% FI) alone did not significantly alter APX activity, the combination of severe drought and AsA treatment revealed a remarkable synergistic effect. Under severe deficit irrigation, 200 and 400 mg L⁻¹ AsA treatments increased APX activity by 64% and 135%, respectively, compared to untreated controls. The maximum enhancement (224.7%) was observed in plants receiving 400 mg L⁻¹ AsA under severe drought conditions compared to untreated plants at 100%FI, highlighting the crucial role of the ascorbate-glutathione cycle in drought adaptation.

SOD activity exhibited a complex response pattern across treatment combinations (Fig. 5F). Under severe deficit irrigation, both AsA concentrations (200 and 400 mg L⁻¹) significantly enhanced SOD activity by approximately 89% compared to untreated stressed plants. This enhanced SOD capacity, responsible for superoxide radical dismutation, represents a first-line defense mechanism that works coordinately with the H₂O₂-scavenging enzymes (CAT and APX) to provide comprehensive ROS protection under drought stress.

### Multivariate analysis reveals a coordinated physiological response to AsA priming

**Fig. 6:**
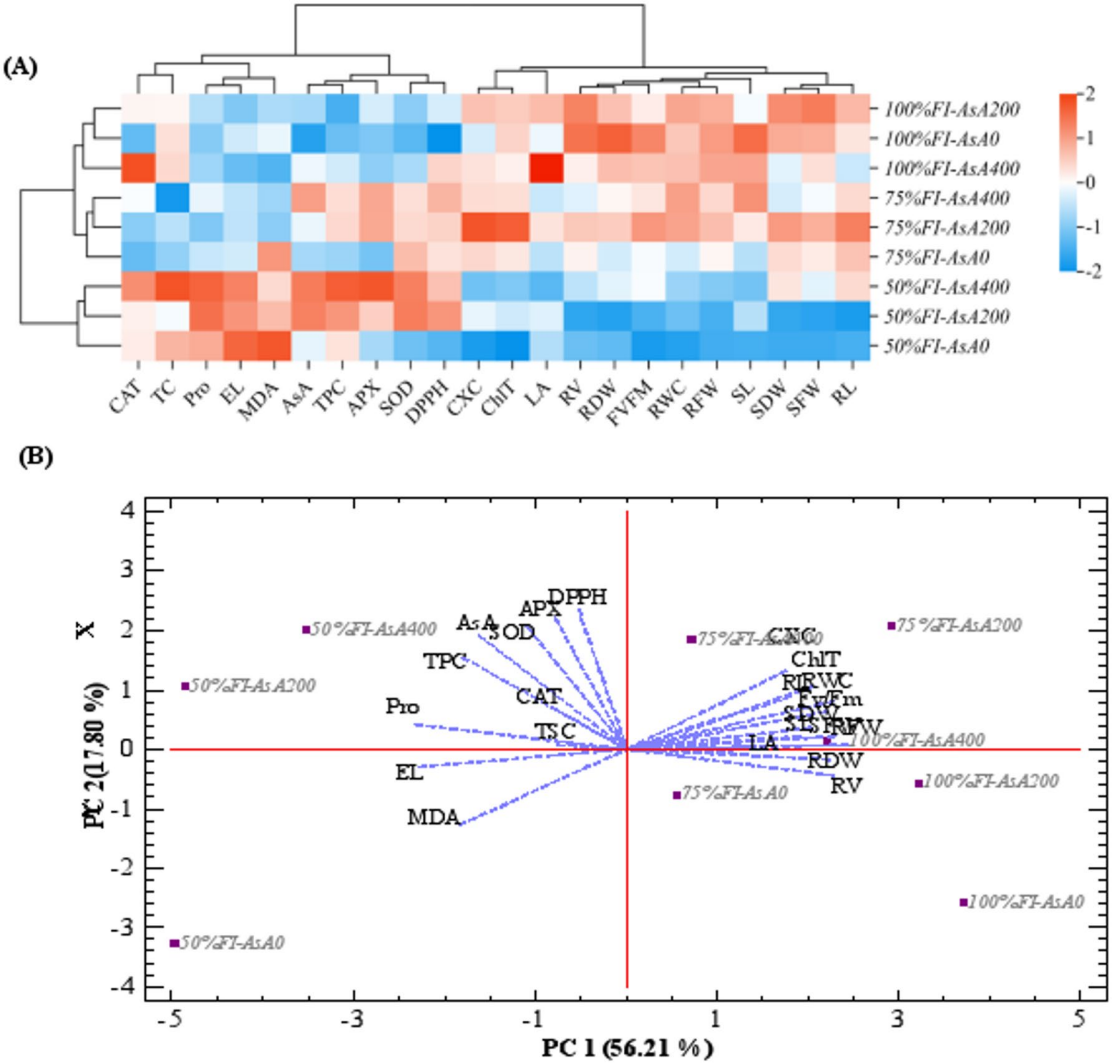
Heatmap and hierarchical clustering (**A**) of different physio-biochemical characteristics that differentially expressed in AsA treated- and untreated-*H. perforatum* plants under drought stress to elucidate the traits relationships across treatments. Principal component analysis (treatment-by-trait biplot) [**B**] of different physio-biochemical characteristics of *H. perforatum* plants under drought stress conditions. The traits are shoot length (SL), shoot fresh weight (SFW), shoot dry weight (SDW), root length (RL), root dry weight (RDW), root fresh weight (RFW), root volume (RV), relative water content (RWC), Fv/Fm index, electrolyte leakage (EL), catalase (CAT), ascorbate peroxidase (APX), superoxide dismutase (SOD), malondialdehyde (MDA), total chlorophyll (ChlT), carotenoid (CXC), ascorbate (AsA), proline (Pro), total soluble carbohydrate content (TSC), total phenolic content (TPC), DPPH radical scavenging. FI: full irrigation; 75% FI: moderate deficit irrigation; and 50% FI: severe deficit irrigation. The color scale displays the intensity of mean values of different characteristics: Blue and red boxes represent significant decrease and increase, respectively.

Multivariate statistical approaches were employed to integrate the comprehensive dataset of morphological, physiological, and biochemical parameters. Hierarchical clustering analysis revealed distinct grouping patterns among the measured variables, forming two primary clusters (Fig. 6A). Cluster A comprised growth-related parameters including root and shoot biomass traits (SFW, RFW, SDW, RDW), leaf area (LA), root and shoot length (SL, RL), root volume (RV), photosynthetic efficiency (Fv/Fm), and relative water content (RWC). Cluster B contained stress-responsive biomarkers: proline, total soluble carbohydrates (TSC), electrolyte leakage (EL), malondialdehyde (MDA), endogenous ascorbate (AsA), antioxidant enzyme activities (CAT, SOD, APX), and total phenolic content (TPC).

The heatmap visualization demonstrated that drought stress consistently reduced the values of Cluster A parameters while simultaneously elevating Cluster B biomarkers. Crucially, exogenous AsA application at 400 mg L⁻¹ under drought conditions partially restored the expression pattern of Cluster A traits toward control levels while modulating the stress response of Cluster B parameters. Specifically, AsA treatment reduced oxidative stress markers (EL, MDA) while further enhancing the accumulation of protective compounds (proline, TSC, TPC) and antioxidant enzymes.

Principal component analysis (PCA) further validated these patterns, with the first two principal components accounting for 74.0% of total variance (PC1: 56.2%; PC2: 17.8%) (Fig. 6B). PC1 showed strong positive loadings with growth parameters (SFW, SDW, RFW, RDW, LA, SL), photosynthetic pigments (ChlT, Cx + c), Fv/Fm, and RWC, effectively representing a “growth and photosynthesis” axis. PC2 was positively associated with antioxidant defense components, including enzyme activities (CAT, SOD, APX), endogenous AsA, TPC, and osmoprotectants (proline, TSC), representing a “stress response” axis.

The PCA biplot clearly separated treatment groups along these physiological axes. Well-watered plants clustered in the positive PC1 region, while drought-stressed plants shifted toward negative PC1 values. Notably, AsA-treated plants under drought stress occupied an intermediate position, with a clear tendency toward the positive PC1 quadrant occupied by control plants. This distribution pattern demonstrates that AsA application partially preserved growth and photosynthetic characteristics while simultaneously enhancing antioxidant capacity under drought conditions.

Untreated plants under severe drought (50% FI) were strongly associated with oxidative stress indicators (EL, MDA) in the negative PC1/positive PC2 quadrant. In contrast, AsA-treated plants under similar stress conditions showed reduced association with stress markers and increased projection toward antioxidant defense compounds and enzymes. This multivariate integration confirms that exogenous AsA fundamentally altered the physiological response strategy to drought, enhancing tolerance mechanisms while mitigating damage pathways.

## Discussion

The present study investigated the efficacy of foliar AsA application in mitigating the effects of moderate and severe drought stress in *H. perforatum*. Our results demonstrate that exogenous AsA significantly influenced the growth, physiological, and biochemical characteristics of *H. perforatum* under drought conditions, which severely impaired plant growth otherwise. This finding aligns with and extends the documented role of AsA as a safe and beneficial molecule for enhancing plant resilience^12,20,42–44^.

Consistent with previous observations in St. John’s Wort^24^, severe deficit irrigation led to a significant reduction in growth parameters, including shoot and root biomass and length. This growth inhibition can be attributed to a combination of factors, including diminished photosynthetic pigment levels, osmotic imbalance, cell dehydration, the generation of ROS, and impaired nutrient uptake^1,45,46^. However, application of AsA ameliorated these effects, as treated plants exhibited greater biomass under drought stress compared to untreated controls (Fig. 1). Furthermore, a concentration of 400 mg L⁻¹ AsA was more effective than 200 mg L⁻¹ in improving several growth traits (Figs. 1 and 2). These findings align with studies on sorghum^44^, wheat^47^, and jute^20^, supporting the role of AsA in alleviating drought stress. AsA is a crucial modulator of plant development, vital for cellular expansion and elongation^15,16^, stimulating cellular proliferation^12^, and inducing the synthesis of growth regulators like auxin^14^. Therefore, our results suggest that exogenous AsA application can effectively mitigate the detrimental impacts of drought on the growth and development of *H. perforatum*.

Osmotic adjustment is a key survival strategy under water stress, primarily mediated by the accumulation of compatible solutes such as proline and soluble carbohydrates^3,48^. In our study, elevated proline content was observed as an adaptive response exclusively under severe drought stress (Fig. 4E), a result consistent with other work on *H. perforatum*^24^. Notably, drought-stressed plants treated with AsA accumulated even higher levels of proline (Fig. 4E), corroborating findings in pepper^18^ and peach^42^. Similarly, the combination of AsA pretreatment and drought stress further increased soluble carbohydrate content (Fig. 4), echoing results from studies on pepper^18^ and oat^11^. The decline in RWC under severe stress (Fig. 4), consistent with the literature^24,42^, was likely less severe in AsA-treated plants due to this enhanced osmotic adjustment. PCA and heatmap data confirmed a strong positive correlation between total soluble carbohydrates, proline content, and AsA-treated drought-stressed plants (Fig. 6). This leads to the conclusion that exogenous AsA enhances the accumulation of osmolytes, thereby improving osmotic adjustment and helping maintain higher RWC under drought conditions. This AsA-enhanced osmotic adjustment, evidenced by elevated proline and soluble carbohydrates, is crucial for maintaining cellular turgor and hydration. By helping to sustain a favorable water potential gradient, it supports continued cell expansion and stomatal function, thereby contributing to the observed preservation of leaf area (Fig. 1A) and shoot growth (Fig. 1B) under drought.

Water deficit induces the generation of ROS, which disrupts cellular membranes and directly degrades photosynthetic pigments and inhibits the Photosystem II (PSII) complex^49^. This dual damage is evidenced in our study by the concurrent decline in total chlorophyll (Fig. 3A) and the maximum quantum efficiency of PSII (Fv/Fm; Fig. 3D) under severe drought. The application of exogenous AsA significantly mitigated the loss of both chlorophyll content and Fv/Fm. This indicates that AsA’s antioxidant action protects the structural integrity of thylakoid membranes and the PSII reaction centers, thereby helping to maintain the core photochemical capacity essential for carbon assimilation and growth under stress.

Plants counteract cytotoxic ROS by upregulating a complex antioxidant system comprising both non-enzymatic and enzymatic components, such CAT, APX, SOD, AsA, carotenoids, and total phenolic compounds^6,49^. These antioxidants are crucial for maintaining redox homeostasis for essential physiological processes^50^. In this study, severe drought stress induced an increase in TPC, endogenous AsA, antioxidant capacity, and carotenoids. The subsequent enhancement of these non-enzymatic antioxidants following exogenous AsA application highlights their significant role in drought adaptation. Phenolic compounds, as secondary metabolites, employ multiple mechanisms to alleviate stress. The higher TPC in AsA-treated, drought-stressed plants suggests a contribution to drought tolerance, a finding supported by similar interactions in calendula^51^ and jute^20^. This indicates a significant synergy between TPC and AsA in sustaining cellular redox homeostasis.

AsA is renowned for its direct antioxidant role in ROS detoxification^52,53^. Our finding that endogenous AsA content increased in response to exogenous application under stress is consistent with previous reports^20,43^. Modulating endogenous AsA levels, through biosynthesis or recycling, is critical for abiotic stress tolerance^53^. The recycling and regeneration of AsA via the Halliwell-Asada pathway (involving AsA and APX) is particularly vital for maintaining redox homeostasis under stress, often more so than the size of the total ascorbate pool itself^54^. Furthermore, drought stress upregulates genes involved in AsA recycling, thereby enhancing drought resistance^12^.

The activities of key antioxidant enzymes (APX, CAT, SOD) responded to drought in a manner dependent on AsA treatment (Fig. 5). SOD activity increased in AsA-treated plants under severe stress compared to controls, a response also documented in rapeseed and pepper under similar conditions^7,18^, though contrasting results exist^11^. The regulation of these enzymes is complex and directly influenced by ROS concentrations^55^. SOD, the first enzyme in the ROS detoxification pathway, converts superoxide into hydrogen peroxide (H₂O₂), which can itself inhibit SOD^56^. Therefore, SOD’s efficiency depends on downstream enzymes like APX and CAT to rapidly remove H₂O₂^33^. CAT is crucial for scavenging H₂O₂, though peroxidases like APX have a higher affinity for it^57^. In our research, exogenous AsA application under drought significantly enhanced the activities of both CAT and APX. A similar pattern of increased CAT and/or peroxidase activities with AsA has been observed in peach^42^, pepper^18^, and jute^20^. The PCA and heatmap analysis further revealed a stronger positive correlation among antioxidant enzymes in AsA-treated plants than in those subjected to drought alone, indicating that AsA upregulates the enzymatic antioxidant system. This coordinated enhancement likely represents a fundamental mechanism to combat oxidative stress and protect against ROS-induced damage.

The multivariate statistical analyses provide a holistic view that powerfully integrates these discrete physiological and biochemical responses. The hierarchical clustering (Fig. 6A) distinctly separated ‘growth and photosynthesis’ parameters (Cluster A) from ‘stress response and defense’ biomarkers (Cluster B). This visual representation confirms that drought stress systematically suppressed Cluster A traits while activating Cluster B. Crucially, the PCA biplot (Fig. 6B) reveals that AsA application fundamentally altered the drought response trajectory. Untreated, drought-stressed plants were positioned in the quadrant associated with oxidative damage (high EL, MDA). In contrast, AsA-treated plants under the same stress shifted towards a profile characterized by enhanced antioxidant capacity (high CAT, APX, SOD, endogenous AsA) and elevated osmotic adjustment (high proline, TSC). This shift correlates with their intermediate position along PC1 (‘growth and photosynthesis’), closer to well-watered controls. Therefore, the PCA confirms that AsA does not merely suppress stress symptoms but promotes a coordinated physiological strategy that balances the maintenance of core metabolic functions with the potentiation of specific protective pathways.

## Conclusions

This study demonstrates that foliar application of ascorbic acid (AsA) effectively enhances drought tolerance in *H. perforatum* through multiple interconnected mechanisms. The optimal concentration of 400 mg L⁻¹ AsA significantly mitigated drought-induced growth inhibition by preserving biomass accumulation and maintaining photosynthetic function. The protective effects were primarily mediated through enhanced osmotic adjustment, evidenced by increased proline and soluble carbohydrate accumulation, which contributed to improved water status maintenance under drought conditions. Furthermore, AsA application orchestrated a comprehensive enhancement of the antioxidant defense system, including both enzymatic components (CAT, APX, SOD) and non-enzymatic antioxidants (endogenous AsA, phenolics), resulting in reduced oxidative damage markers (MDA and electrolyte leakage). The multivariate analysis confirmed that AsA treatment shifted the plant’s response strategy from stress damage toward stress adaptation by simultaneously maintaining growth parameters and enhancing defense mechanisms. These findings suggest that exogenous AsA application represents a practical and effective approach for improving drought tolerance in this valuable medicinal species, potentially through priming of defense systems before stress occurrence. Future research should focus on elucidating the molecular mechanisms underlying AsA-mediated priming effects and validating these findings under field conditions to facilitate commercial application.

## Supplementary Information

Below is the link to the electronic supplementary material.

Supplementary Material 1

## Data Availability

The datasets generated and analyzed during the current study are available from the corresponding author (N.E.) upon reasonable request.

## References

[1] Cohen, I., Zandalinas, S. I., Huck, C., Fritschi, F. B. & Mittler, R. Meta-analysis of drought and heat stress combination impact on crop yield and yield components. *Physiol. Plant.* **171**, 66–76. 10.1111/ppl.13203 (2021). 32880977 10.1111/ppl.13203

[2] Gu, Z. et al. Role of microbes in alleviating crop drought stress: A review. *Plants* **13**, 384. 10.3390/plants13030384 (2024). 38337917 10.3390/plants13030384 PMC10857462

[3] Naikwade, P. V. Plant responses to drought stress: Morphological, physiological, molecular approaches, and drought resistance. In Plant Metabolites Under Environmental Stress (149–183). Apple Academic. (2023).

[4] Osakabe, Y., Osakabe, K., Shinozaki, K. & Tran, L. S. P. Response of plants to water stress. *Front. Plant. Sci.* **5**, 86. 10.3389/fpls.2014.00086 (2014). 24659993 10.3389/fpls.2014.00086 PMC3952189

[5] Celi, G. E. A., Gratão, P. L., Lanza, M. G. D. B. & Dos Reis, A. R. Physiological and biochemical roles of ascorbic acid on mitigation of abiotic stresses in plants. *Plant. Physiol. Biochem.* **107970**10.1016/j.plaphy.2023.107970 (2023). 10.1016/j.plaphy.2023.107970 37625254

[6] Mansoor, S. et al. Reactive oxygen species in plants: from source to sink. *Antioxidants* **11**, 225. 10.3390/antiox11020225 (2022). 35204108 10.3390/antiox11020225 PMC8868209

[7] Hasanuzzaman, M. et al. Foliar application of ascorbic acid and Tocopherol in conferring salt tolerance in rapeseed by enhancing K+/Na + homeostasis, osmoregulation, antioxidant defense, and glyoxalase system. *Agronomy* **13**, 361. 10.3390/agronomy13020361 (2023).

[8] Hasanuzzaman, M. et al. Reactive oxygen species and antioxidant defense in plants under abiotic stress: revisiting the crucial role of a universal defense regulator. *Antioxidants* **9**, 681. 10.3390/antiox9080681 (2020). 32751256 10.3390/antiox9080681 PMC7465626

[9] Li, Y. & Ma, Z. Antioxidants and reactive oxygen species (ROS) scavenging enzymes. In: (eds Gao, K., Hutchins, D. A. & Beardall, J.) Research Methods of Environmental Physiology in Aquatic Sciences. Springer, Singapore. 85–91. 10.1007/978-981-15-5354-7_10 (2021).

[10] Shehzad, J. & Mustafa, G. Mechanism of reactive oxygen species regulation in plants. In Reactive Oxygen Species: Prospects in Plant Metabolism (17–41). Singapore: Springer Nature Singapore. (2023).

[11] El-Beltagi, H. S., Shah, S., Ullah, S., Sulaiman Mansour, A. T. & Shalaby, T. A. Impacts of ascorbic acid and alpha-tocopherol on Chickpea (*Cicer arietinum* L.) grown in water deficit regimes for sustainable production. *Sustainability* **14**, 8861. 10.3390/su14148861 (2022).

[12] Gallie, D. R. L-Ascorbic acid: a multifunctional molecule supporting plant growth and development. Scientifica. 2013: 795964. (2013). 10.1155/2013/795964 10.1155/2013/795964 PMC3820358 24278786

[13] MacDonald, M. T., Kannan, R. & Jayaseelan, R. Ascorbic acid preconditioning effect on broccoli seedling growth and photosynthesis under drought stress. *Plants* **11**, 1324. 10.3390/plants11101324 (2022). 35631749 10.3390/plants11101324 PMC9145904

[14] Smirnoff, N. Ascorbic acid metabolism and functions: A comparison of plants and mammals. Free radi. *Biol. Med.* **122**, 116–129. 10.1016/j.freeradbiomed.2018.03.033 (2018). 10.1016/j.freeradbiomed.2018.03.033 PMC6191929 29567393

[15] Paciolla, C. et al. Vitamin C in plants: from functions to biofortification. *Antioxidants* **8**, 519. 10.3390/antiox8110519 (2019). 31671820 10.3390/antiox8110519 PMC6912510

[16] Wu, S. Y. et al. Ascorbic acid-mediated reactive oxygen species homeostasis modulates the switch from tapetal cell division to cell differentiation in Arabidopsis. *Plant. Cell.* **35**, 1474–1495. 10.1093/plcell/koad037 (2023). 36781400 10.1093/plcell/koad037 PMC10118275

[17] Akram, N. A., Shafiq, F. & Ashraf, M. Ascorbic acid-a potential oxidant scavenger and its role in plant development and abiotic stress tolerance. *Front. Plant. Sci.* **8**, 1–17. 10.3389/fpls.2017.00613 (2017). 28491070 10.3389/fpls.2017.00613 PMC5405147

[18] Khazaei, Z., Esmaielpour, B. & Estaji, A. Ameliorative effects of ascorbic acid on tolerance to drought stress on pepper (*Capsicum annuum* L) plants. *Physiol. Mol. Biol. Plants*. **26**, 1649–1662. 10.1007/s12298-020-00846-7 (2020). 32801493 10.1007/s12298-020-00846-7 PMC7415064

[19] Farooq, A. et al. Exogenously applied ascorbic acid-mediated changes in osmoprotection and oxidative defense system enhanced water stress tolerance in different cultivars of safflower (*Carthamus tinctorious* L). *Plants* **9**, 1–15. 10.3390/plants9010104 (2020). 10.3390/plants9010104 PMC7020178 31947709

[20] Sharma, L. et al. Exogenous ascorbic acid application ameliorates drought stress through improvement in morpho-physiology, nutrient dynamics, stress metabolite production and antioxidant activities recovering cellulosic fibre production in jute (*Corchorus olitorius* L). *Ind. Crops Prod.* **217**, 118808 (2024).

[21] Barnes, J., Anderson, L. A. & Phillipson, J. D. St john’s wort (*Hypericum perforatum* L.): a review of its chemistry, Pharmacology and clinical properties. *J. Pharm. Pharmacol.* **53**, 583–600. 10.1211/0022357011775910 (2001). 11370698 10.1211/0022357011775910

[22] İrem, A. & Yüksel, K. Examination of some quality analysis carried out to the determination of standardization of St. John’s wort (*Hypericum perforatum* L.) oil used in traditional medicine applications. *Biol. Divers. Conserv.* **15**, 249–255. 10.46309/biodicon.2022.1120535 (2022).

[23] Zobayed, S. M. A., Afreen, F. & Kozai, T. Phytochemical and physiological changes in the leaves of St. John’s wort plants under a water stress condition. *Environ. Exp. Bot.* **59**, 109–116. 10.1016/j.envexpbot.2005.10.002 (2007).

[24] Torun, H., Eroglu, E., Yalcin, V. & Usta, E. U. Physicochemical and antioxidant responses of St. John’s wort (*Hypericum perforatum* L.) under drought stress. *J. Sci. Technol.* **9**, 40–50. 10.29130/dubited.847860 (2021).

[25] Gaafar, A. A. et al. Ascorbic acid induces the increase of secondary metabolites, antioxidant activity, growth, and productivity of the common bean under water stress conditions. *Plants* **9**, 627. 10.3390/plants9050627 (2020). 32423048 10.3390/plants9050627 PMC7285268

[26] Allen, R., Pereira, L. S., Raes, D. & Smith, M. Crop Evapotranspiration (Guidelines for computing crop water requirements. FAO Irrigation and Drainage Paper,No. 56.FAO, Rome, Italy (1998).

[27] Hosseini, F., Mosaddeghi, M. R., Dexter, A. R. & Sepehri, M. Maize water status and physiological traits as affected by root endophytic fungus Piriformospora indica under combined drought and mechanical stresses. *Planta* **247** (5), 1229–1245. 10.1007/s00425-018-2861-6 (2018). 29453661 10.1007/s00425-018-2861-6

[28] Shirani Bidabadi, S., Dehghanipoodeh, S. & Wright, G. C. Vermicompost leachate reduces some negative effects of salt stress in pomegranate. *Int. J. Recycl Org. Waste Agricult*. **6** (3), 255–263 (2017).

[29] Smart, R. E. & Bingham, G. E. Rapid estimates of relative water content. *J. Plant. Physiol.* **53**, 258–260. 10.1104/pp.53.2.258 (1974). 10.1104/pp.53.2.258 PMC541374 16658686

[30] Lichtenthaler, H. K. & Buschmann, C. Chlorophylls and carotenoids: measurement and characterization by UV-VIS spectroscopy. In: Current protocols in food analytical chemistry. F4.2.1–F4.2.6. (2001). 10.1002/0471142913.faf0403s01

[31] Maxwell, K. & Johnson, G. Chlorophyll fuorescence—a practical guide. *J. Exp. Bot.* **345**, 659–668. 10.1093/jxb/51.345.659 (2000). 10.1093/jxb/51.345.659 10938857

[32] Lutts, S., Kinet, J. M. & Bouharmont, J. NaCl-induced senescence in leaves of rice (*Oryza sativa* L.) cultivars differing in salinity resistance. *Ann. Bot.* **78**, 389–398. 10.1006/anbo.1996.0134 (1996).

[33] Amirikhah, R., Etemadi, N., Sabzalian, M. R., Nikbakht, A. & Eskandari, A. Gamma radiation negatively impacted seed germination, seedling growth and antioxidant enzymes activities in tall fescue infected with Epichloë endophyte. *Ecotoxicol. Environ. Saf.* **216**, 112169. 10.1016/j.ecoenv.2021.112169 (2021). 33826977 10.1016/j.ecoenv.2021.112169

[34] Bates, L. S., Waldren, R. P. A. & Teare, I. D. Rapid determination of free proline for water-stress studies. *Plant. soil.* **39** (1), 205–207. 10.1007/BF00018060 (1973).

[35] Rastegari, S., Alavi, N., Mohayeji, S. M. & M Effect of Salicylic acid and Pre-Cold treatment on flower induction in saffron. *Scientifica* **2022** (1), 6108161. 10.1155/2022/6108161 (2022). 36311284 10.1155/2022/6108161 PMC9616660

[36] Mukherjee, S. P. & Choudhuri, M. A. Implications of water stress-induced changes in the levels of endogenous ascorbic acid and hydrogen peroxide in vigna seedlings. *Physiol. Plant.* **58**, 166–170. 10.1111/j.1399-3054.1983.tb04162.x (1983).

[37] Julkunen-Tiitto, R. Phenolic constituents in the leaves of Northern willows: methods for the analysis of certain phenolics. *J. Agric. Food Chem.* **33**, 213–217. 10.1021/jf00062a013 (1985).

[38] Alghanem, S. Allelopathic effects of Aizoon canariense leaf leachates on growth, biochemistry, and oxidative stress responses in selected crop species. *Front. Plant. Sci.* **16**, 1659978. 10.3389/fpls.2025.1659978 (2025). 40963821 10.3389/fpls.2025.1659978 PMC12436394

[39] Aebi, H. E. Catalase. In: Bergmeyer HU (Ed.), Methods of Enzymatic Analysis.Verlag Chemie, Weinheim, pp. 273–286. (1983). 10.1016/B978-0-12-091302-2.50032-3

[40] Nakano, Y. & Asada, K. Hydrogen peroxide is scavenged by ascorbate-specific peroxidase in spinach Chloroplast. *Plant. Cell. Physiol.* **22**, 867–880. 10.1093/oxfordjournals.pcp.a076232 (1981).

[41] Giannopolitis, C. N. & Ries, S. K. Superoxide dismutase: occurrence in higher plants. *Plant. Physiol.* **59**, 309–314. 10.1104/pp.59.2.309 (1977). 16659839 10.1104/pp.59.2.309 PMC542387

[42] Penella, C., Calatayud, Á. & Melgar, J. C. Ascorbic acid alleviates water stress in young Peach trees and improves their performance after rewatering. *Front. Plant. Sci.* **8**, 1627. 10.3389/fpls.2017.01627 (2017). 28979284 10.3389/fpls.2017.01627 PMC5611396

[43] Alamri, S. A. et al. Ascorbic acid improves the tolerance of wheat plants to lead toxicity. *J. Plant. Interact.* **13**, 409–419. 10.1080/17429145.2018.1491067 (2018).

[44] Elsiddig, A. M. I., Zhou, G., Nimir, N. E. A. & Ali, A. Y. A. Effect of exogenous ascorbic acid on two sorghum varieties under different types of salt stress. *Chil. J. Agric. Res.* **82**, 10–20. 10.4067/S0718-58392022000100010 (2021).

[45] Ahmad, R., Alsahli, A. A., Alansi, S. & Altaf, M. A. Exogenous melatonin confers drought stress by promoting plant growth, photosynthetic efficiency and antioxidant defense system of pea (*Pisum sativum* L). *Sci. Hortic.* **322**, 112431. 10.1016/j.scienta.2023.112431 (2023).

[46] Forni, C., Duca, D. & Glick, B. R. Mechanisms of plant response to salt and drought stress and their alteration by rhizobacteria. *Plant. Soil.* **410**, 335–356. 10.1007/s11104-016-3007-x (2017).

[47] Athar, H., Khan, A. & Ashraf, M. Exogenously applied ascorbic acid alleviates salt induced oxidative stress in wheat. *Environ. Exp. Bot.* **63**, 224–231. 10.1016/j.envexpbot.2007.10.018 (2008).

[48] Ashraf, M. & Foolad, M. R. Roles of Glycine betaine and proline in improving plant abiotic stress resistance. *Environ. Exp. Bot.* **59**, 206–216. 10.1016/j.envexpbot.2005.12.006 (2007).

[49] Gill, S. S. & Tuteja, N. Reactive oxygen species and antioxidant machinery in abiotic stress tolerance in crop plants. *Plant. Physiol. Biochem.* **48**, 909–930. 10.1016/j.plaphy.2010.08.016 (2010). 20870416 10.1016/j.plaphy.2010.08.016

[50] Saed-Moucheshi, A., Shekoofa, A. & Pessarakli, M. Reactive oxygen species (ROS) generation and detoxifying in plants. *J. Plant. Nutr.* **37**, 1573–1585. 10.1080/01904167.2013.868483 (2014).

[51] Azizi, F., Amiri, H. & Ismaili, A. Effect of drought stress and ascorbic acid on some morphological and biochemical traits of *Calendula officinalis* L. *J. Plant. Process. Function*. **10** (44), 11–22 (2021).

[52] Padh, H. Cellular functions of ascorbic acid. *Biochem. Cell. Biol.* **68**, 1166–1173. 10.1139/o90-173 (1990). 2268411 10.1139/o90-173

[53] Xiao, M. et al. The multiple roles of ascorbate in the abiotic stress response of plants: antioxidant, cofactor, and regulator. *Front. Plant. Sci.* **12**, 598173. 10.3389/fpls.2021.598173 (2021). 33912200 10.3389/fpls.2021.598173 PMC8072462

[54] Chen, Z. & Gallie, D. R. The ascorbic acid redox state controls guard cell signaling and stomatal movement. *Plant. Cell.* **16**, 1143–1162. 10.1105/tpc.021584 (2004). 15084716 10.1105/tpc.021584 PMC423206

[55] Gudkov, S. V., Grinberg, M. A., Sukhov, V. & Vodeneev, V. Effect of ionizing radiation on physiological and molecular processes in plants. *J. Environ. Radioact*. **202**, 8–24. 10.1016/j.jenvrad.2019.02.001 (2019). 30772632 10.1016/j.jenvrad.2019.02.001

[56] Casano, L. M., Gomes, L. D., Lascano, H. R., Gonzales, C. A. & Trippi, V. S. Inactivation and degradation of CuZn-SOD by active oxygen species in wheat chloroplasts exposed to photooxidative stress. *Plant. Cell. Physiol.* **38**, 433–440. 10.1093/oxfordjournals.pcp.a029186 (1997). 9177029 10.1093/oxfordjournals.pcp.a029186

[57] Bela, K., Horvath, E., Szabados, L., Tari, I. & Csiszar, J. Plant glutathione peroxidases: emerging role of the antioxidant enzymes in plant development and stress responses. *J. Plant. Physiol.* **176**, 192–201. 10.1016/j.jplph.2014.12.014 (2015). 25638402 10.1016/j.jplph.2014.12.014

