# Supplementary figures and images for: Exogenous ascorbic acid enhances drought tolerance in Hypericum perforatum L. by modulating antioxidant defense and osmotic adjustment
Source: Sci Rep. 2026 Jan 31;16:6822. doi: 10.1038/s41598-026-35931-6 (PMC12917152; doi:10.1038/s41598-026-35931-6)

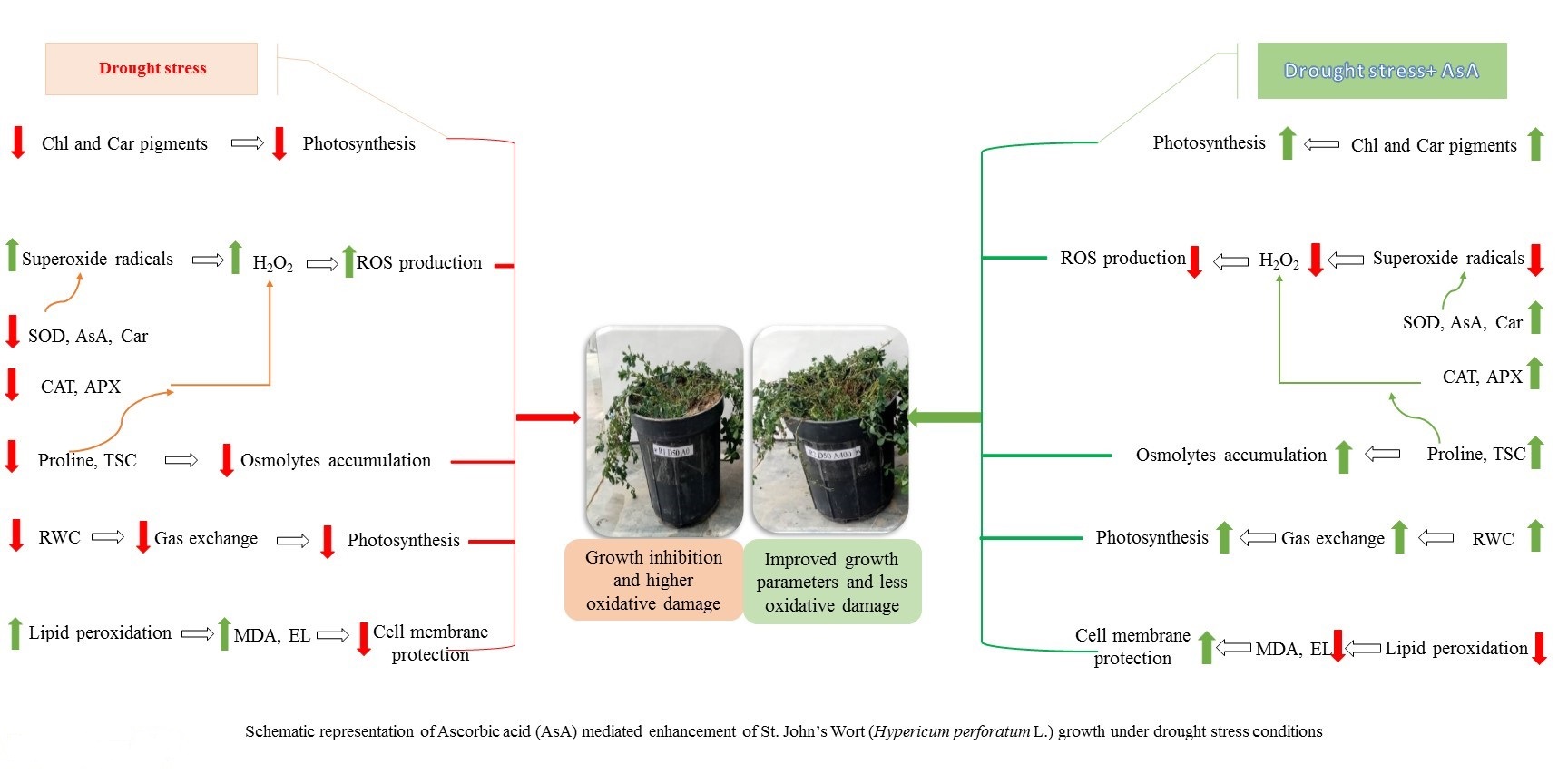

Supplement: Supplementary file 1 — Supplementary Material 1 [file 41598_2026_35931_MOESM1_ESM.jpg]
